# A bioenergetic assessment of photosynthetic growth of *Synechocystis* sp. PCC 6803 in continuous cultures

**DOI:** 10.1186/s13068-015-0319-7

**Published:** 2015-09-04

**Authors:** Eleftherios Touloupakis, Bernardo Cicchi, Giuseppe Torzillo

**Affiliations:** Istituto per lo Studio degli Ecosistemi, CNR, Sede di Firenze, Via Madonna del Piano, 10, 50019 Sesto Fiorentino, Italy

**Keywords:** Photobioreactor, Continuous culture, Cyanobacteria, Light conversion efficiency (LCE), Fluorescence

## Abstract

**Background:**

*Synechocystis* sp. PCC 6803, a model organism used for bioenergy and bioplastic production, was grown in continuous culture to assess its most important bioenergetic parameters.

**Results:**

Biomass yield on light energy of 1.237 g mol photons^−1^ and maintenance energy requirement of 0.00312 mol photons g^−1^ h^−1^ were calculated. This corresponded to a light conversion efficiency of 12.5 %, based on the model of Pirt which was about 35 % lower than the theoretical one based on the stoichiometric equation for the formation of biomass on carbon dioxide, water, and nitrate. The maximum *F*_v_/*F*_m_ ratio recorded in the *Synechocystis* cultures was 0.57; it progressively declined to 0.45 as the dilution rate increased. An over-reduction of reaction centers at a high dilution rate was also recorded, together with an increased V_J_ phase for the chlorophyll fluorescence transient. In contrast, the chlorophyll optical cross section increased by about 40 % at the fastest dilution rate, and compensated for the decline in *F*_v_/*F*_m_, thus resulting in a constant total photosynthesis rate (photosynthesis plus respiration). Chlorophyll content was maximum at the lowest dilution rate and was 48 % lower at the highest one, while phycocyanin, and total carotenoids decreased by about 42 % and 37 %, respectively. Carotenoid analysis revealed increased echinenone, zeaxanthin, and myxoxanthophyll contents as the dilution rate increased (40.6, 63.8 and 35.5 %, respectively, at the fastest dilution rate). A biochemical analysis of the biomass harvested at each different dilution rates showed no changes in the lipid content (averaging 11.2 ± 0.6 % of the dry weight), while the protein content decreased as the dilution rate increased, ranging between 60.7 ± 1.1 and 72.6 ± 0.6 %. Amino acids pattern did not vary with the dilution rate. Carbohydrate content ranged from 9.4 to 16.2 % with a mean value of 11.2 ± 1.4 %.

**Conclusions:**

The present work provides useful information on the threshold of light conversion efficiency in *Synechocystis*, as well as basic bioenergetic parameters that will be helpful for future studies related to its genetic transformation and metabolic network reconstruction.

**Electronic supplementary material:**

The online version of this article (doi:10.1186/s13068-015-0319-7) contains supplementary material, which is available to authorized users.

## Background

The cyanobacterium *Synechocystis* sp PCC 6803 (hereafter *Synechocystis*) is widely used as a model organism for the study of photosynthetic processes, since it can easily be transformed and is well characterized. Its genome is completely sequenced, and a variety of mutants is available. The use of *Synechocystis* has been proposed for the production of biofuels and also for that of chemicals and biomaterials [1–3]. Recent studies have demonstrated that *Synechocystis* is a good candidate organism for photobiological hydrogen production [4]. It has been genetically engineered for the photosynthetic production of isoprene hydrocarbon currently used as feedstock in the synthetic chemistry industry for the production of commercial commodities [5–7].

Understanding the dependence of *Synechocystis* growth on light absorption is a pre-requisite for achieving (a) high photosynthetic efficiency, (b) future genetic manipulation and (c) scaling-up of cultures outdoors. For these purposes, continuous cultures operated at steady state present several advantages, including constant growth rates and a constant biomass composition for extended time periods [8]. Indeed, whether *Synechocystis* is a wild type or a genetically modified strain, the enhancement of its productivity will depend on identifying constraints on its growth and upon overcoming those limitations with well-designed photobioreactors (PBR) [9, 10].

In this study, we used a flat-bed PBR to assess the bioenergetics of photosynthetic growth of *Synechocystis* in continuous cultures. To this end, the cultures were subjected to different dilution rates, which established conditions ranging from light limitation to light saturation.

## Results

### Culture characterization

**Fig. 1:**
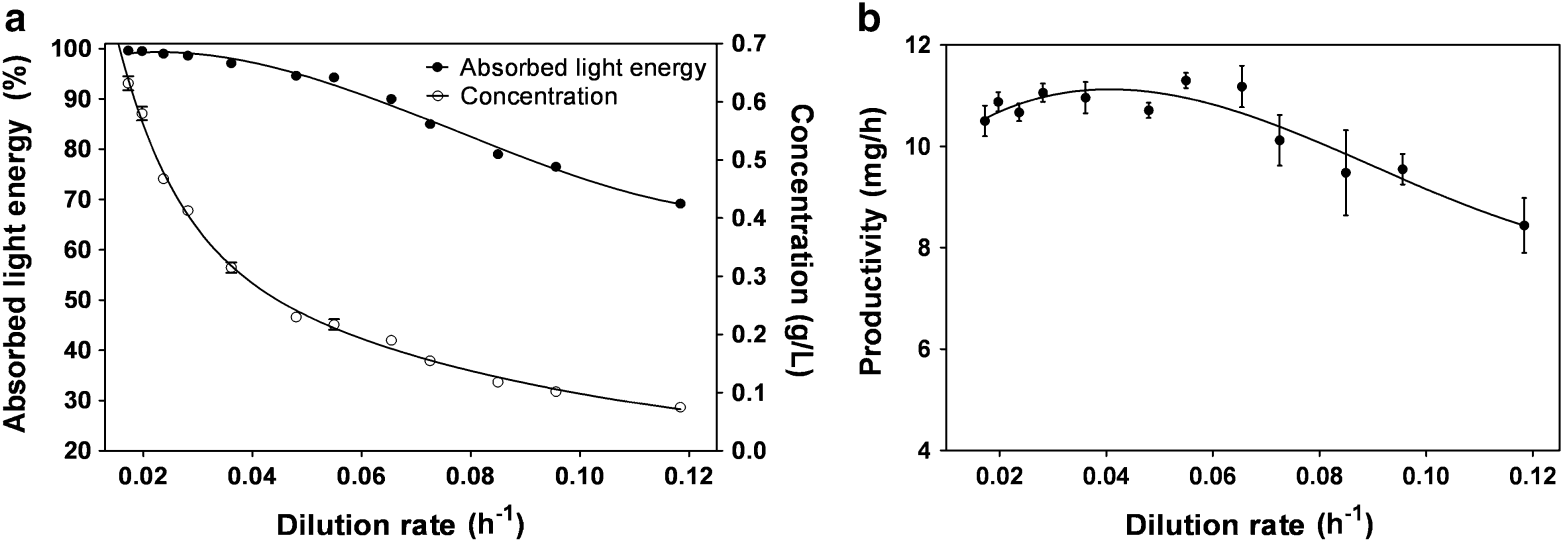
**a** Relationship between light energy absorption, as a function of the dilution rate; **b** changes in productivity as a function of the dilution rate during the *Synechocystis* cultivations. Data are the average from at least three measurements; *error bars* represent the standard deviations

The dependence of cell concentration on the dilution rate (*D*) is shown in Fig. 1a. The maximum cell concentration of the culture at the steady state was 632 ± 12 mg L^−1^, and it decreased to 75 ± 0 mg L^−1^ at the highest *D*. Productivity values at different *D* are shown in Fig. 1b and Table 1. The highest culture productivity was 11.30 ± 0.15 mg h^−1^, recorded at *D* = 0.055 h^−1^, while the lowest value of productivity, 8.44 ± 0.54 mg h^−1^, was found at *D* = 0.1184 h^−1^, close to wash-out. It was, therefore, assumed to be the maximum growth rate of the culture, corresponding to a minimum doubling time of 5.8 h (Table 1; Fig. 1).

**Table 1:** Cell concentration, light absorption, productivity, growth yield, actual biomass yield on light energy (*Y*) and light conversion efficiency (LCE) of *Synechocystis* in a continuous culture

*D* (h^−1^)	Concentration (mg L^−1^)	Absorbed light (%)	Productivity (mg h^−1^)	*Y*_kJ_ (g kJ^−1^×10^−3^)	*Y*_kJ_ (g kJ^−1^×10^−3^)^a^	*Y* (g mol photons^−1^)	LCE (%)	LCE (%)^a^
0.0173	632 ± 12	99.6	10.50 ± 0.30	4.71 ± 0.13	4.69 ± 0.28	1.02 ± 0.02	10.7 ± 0.5	10.5 ± 0.6
0.0198	580 ± 10	99.5	10.88 ± 0.19	4.89 ± 0.08	4.86 ± 0.21	1.07 ± 0.02	10.7 ± 0.3	10.6 ± 0.4
0.0237	468 ± 3	99.0	10.67 ± 0.17	4.82 ± 0.17	4.77 ± 0.17	1.04 ± 0.01	11.4 ± 0.5	11.3 ± 0.5
0.0282	413 ± 7	98.6	11.06 ± 0.18	5.02 ± 0.09	4.94 ± 0.55	1.09 ± 0.02	11.1 ± 0.7	10.9 ± 1.2
0.0361	315 ± 9	97.1	10.96 ± 0.31	5.05 ± 0.10	4.90 ± 0.10	1.08 ± 0.03	11.9 ± 0.2	11.6 ± 0.2
0.0480	230 ± 3	94.6	10.71 ± 0.15	5.06 ± 0.07	4.79 ± 0.25	1.08 ± 0.01	11.0 ± 0.6	10.3 ± 0.7
0.0550	217 ± 9	94.3	11.30 ± 0.15	5.36 ± 0.13	5.05 ± 0.12	1.17 ± 0.05	11.9 ± 0.3	11.1 ± 0.3
0.0654	190 ± 0	90.0	11.18 ± 0.41	5.55 ± 0.33	5.00 ± 0.49	1.21 ± 0.01	11.6 ± 0.9	10.4 ± 1.0
0.0725	155 ± 7	85.0	10.12 ± 0.50	5.32 ± 0.25	4.52 ± 0.21	1.22 ± 0.05	11.7 ± 0.6	9.9 ± 0.5
0.0849	118 ± 5	79.0	9.48 ± 0.84	5.36 ± 0.29	4.24 ± 0.23	1.17 ± 0.05	11.2 ± 0.6	8.9 ± 0.5
0.0956	102 ± 3	76.5	9.55 ± 0.30	5.58 ± 0.26	4.27 ± 0.20	1.18 ± 0.03	11.4 ± 0.4	8.5 ± 0.3
0.1184	75 ± 0	69.2	8.44 ± 0.54	5.45 ± 0.12	3.77 ± 0.08	1.19 ± 0.01	11.2 ± 0.2	7.7 ± 0.2

Values are mean ± standard deviations calculated during the steady state at each dilution rate

^a^When considering total light absorption

The growth yield *Y*_kJ_ (the amount of dry biomass synthesized per kJ of light energy absorbed) was rather constant along the dilution rate with a mean value of 5.18 ± 0.29 mg kJ^−1^ (Table 1).

By multiplying *Y*_kJ_ values, attained at each *D*, by the heat of combustion of the biomass, it was possible to estimate the apparent light conversion efficiency (LCE). The average heat of combustion of the biomass was 21.98 ± 1.05 kJ g^−1^. The mean LCE resulted close to 11.32 ± 0.41 % at all the *D* tested (Table 1).

### Fluorescence and photosynthetic parameters

The maximum quantum yield of PSII was measured at the various *D*; *F*_v_/*F*_m_ decreased as *D* increased (Additional file 1: Figure S1). This reflects the fact that, at the highest *D*, the cells experienced higher light exposure, which led to a down-regulation of PSII of about 21.5 %. The highest value of *F*_v_/*F*_m_ recorded in this study was 0.572 (Table 2).

**Table 2:** Maximum quantum efficiency of PSII photochemistry (*F*_v_/*F*_m_), effective photochemical quantum yield of PSII (Δ*F*/*F*_m_^′^), effective efficiency of PSII photochemistry (*F*_v_^′^/*F*_m_^′^), non-photochemical quenching (NPQ), photochemical quenching (qP), and quantum yield of CO_2_ assimilation (*Φ*_CO2_) of *Synechocystis* in a continuous culture

*D* (h^−1^)	*F*_v_/*F*_m_	Δ*F*/*F*_m_^′^	*F*_v_^′^/*F*_m_^′^	NPQ	QP	*Φ*_CO2_
0.0173	0.572 ± 0.006	0.464 ± 0.009	0.551 ± 0.001	0.10 ± 0.02	0.84 ± 0.01	0.058 ± 0.001
0.0198	0.564 ± 0.008	0.485 ± 0.001	0.548 ± 0.001	0.06 ± 0.02	0.89 ± 0.02	0.060 ± 0.000
0.0237	0.572 ± 0.013	0.474 ± 0.008	0.555 ± 0.002	0.11 ± 0.01	0.86 ± 0.03	0.059 ± 0.001
0.0282	0.549 ± 0.004	0.458 ± 0.003	0.522 ± 0.006	0.11 ± 0.01	0.88 ± 0.01	0.057 ± 0.001
0.0361	0.525 ± 0.011	0.451 ± 0.002	0.501 ± 0.007	0.06 ± 0.05	0.89 ± 0.16	0.056 ± 0.002
0.0480	0.514 ± 0.010	0.443 ± 0.017	0.493 ± 0.004	0.10 ± 0.03	0.90 ± 0.06	0.055 ± 0.000
0.0550	0.510 ± 0.004	0.411 ± 0.001	0.492 ± 0.006	0.11 ± 0.02	0.83 ± 0.14	0.051 ± 0.000
0.0654	0.506 ± 0.016	0.417 ± 0.001	0.467 ± 0.001	0.11 ± 0.00	0.89 ± 0.04	0.052 ± 0.000
0.0725	0.483 ± 0.016	0.400 ± 0.003	0.468 ± 0.001	0.07 ± 0.00	0.87 ± 0.04	0.050 ± 0.000
0.0849	0.476 ± 0.000	0.390 ± 0.001	0.448 ± 0.000	0.11 ± 0.00	0.87 ± 0.01	0.048 ± 0.000
0.0956	0.452 ± 0.011	0.388 ± 0.012	0.434 ± 0.001	0.08 ± 0.02	0.90 ± 0.02	0.048 ± 0.001
0.1184	0.450 ± 0.001	0.371 ± 0.000	0.433 ± 0.001	0.08 ± 0.02	0.84 ± 0.09	0.046 ± 0.000

Values are mean ± standard deviations calculated over the steady state for each dilution rate

The fluorescence, *F*_s_, and the maximum fluorescence, *F*_m_^′^, i.e., in the light-adapted state, were monitored during the steady state of the culture, and the effective photochemical quantum yield of PSII (Δ*F*/*F*_m_^′^) was determined by performing in situ measurements under illumination. The effective quantum yield of PSII (Δ*F*/*F*_m_^′^) decreased as *D* increased (increment in light exposure per cell), following the same trend as the *F*_v_/*F*_m_ ratio. The overall decrease in the Δ*F*/*F*_m_^′^ of the cells was 23.5 % (Table 2).

The mean value of *Φ*_CO2_ was 0.0533 ± 0.0047 (mol CO_2_ mol photons^−1^) with a difference between the lowest and highest dilution rates of about 20 % (Table 2).

NPQ values of chlorophyll fluorescence were found to be very low at all *D*, indicating a low level of energy dissipation. Accordingly, the qP values were relatively high, constantly between 0.8 and 0.9, indicating that the fraction of PSII reaction centers that were open and capable of photochemistry was high in all the tested dilution rates.

**Fig. 2:**
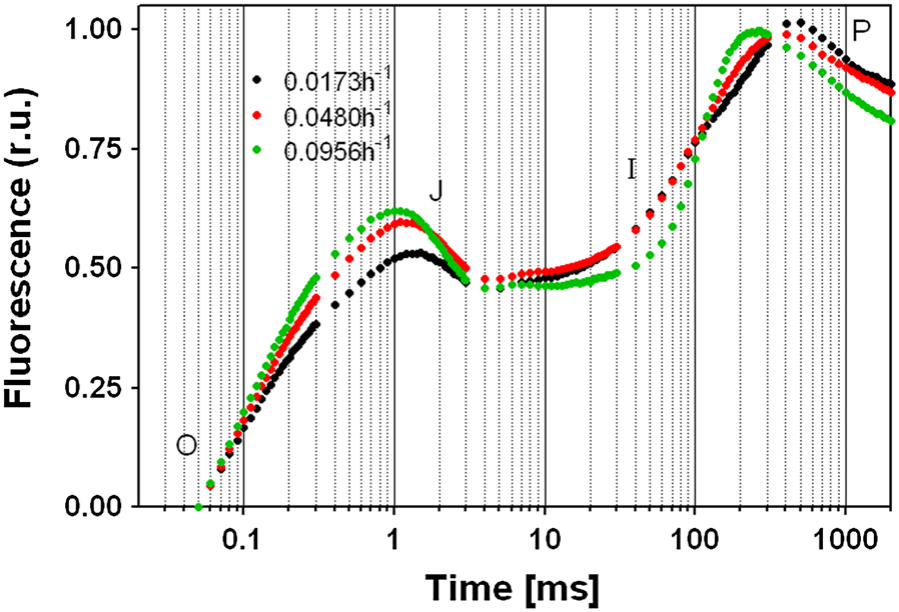
Effect of the dilution rates on chlorophyll a fluorescence transients. Initial fluorescence values were set at zero, and fluorescence values were normalized to maximum fluorescence

Chlorophyll fluorescence-induction kinetics (OJIP) were measured in each experiment. The transient followed the typical polyphasic OJIP rise (Fig. 2). At higher *D* values *M*_0_ and *V*_J_ values increased, indicating a higher rate of closure of the reaction centers and an increment in the net rate of *Q*_A_ reduction (Additional file 2: Table S1). The values of *Ψ*_0_ (probability that a trapped electron can move further ahead than *Q*_A_^−^) and *Φ*_E0_ diminished by 25 and 43 % compared to the lowest *D* values, respectively, evidencing a reduction in the efficiency of PSII to perform photosynthesis, which caused the observed reduction in culture productivity (Additional file 2: Table S1). At increasing *D* values, *S*_m_ decreased, overall by 10 %, indicating a decrease in the size of functional PQ pool.

The overall linear electron transport rate (ETR) was also determined at the steady state of the culture in each experiment (Table 3). The highest ETR value, 0.425 ± 0.001 μmol e^−^ mg chl^−1^ s^−1^, was found at *D* = 0.0198 h^−1^, and this value decreased by 18 % at the highest dilution rate.

**Table 3:** Chlorophyll optical absorption cross-section (a*), maximum electron transfer rate (ETR_max_), O_2_ evolution and respiration rates of *Synechocystis* in a continuous culture

*D* (h^−1^)	Net O_2_ evolution (μmol O_2_ mg chl^−1^ h^−1^)	Respiration (μmol O_2_ mg chl^−1^ h^−1^)	a* (m^2^ mg chl^−1^)	ETR (μmol e^−^ mg chl^−1^ s^−1^)
0.0173	299 ± 6 (314 ± 7)	15 ± 1	0.0112 ± 0.0003	0.386 ± 0.007
0.0198	329 ± 1 (349 ± 3)	20 ± 2	0.0118 ± 0.0005	0.425 ± 0.001
0.0237	322 ± 5 (361 ± 6)	38 ± 1	0.0118 ± 0.0005	0.416 ± 0.007
0.0282	313 ± 2 (359 ± 4)	46 ± 2	0.0120 ± 0.0002	0.404 ± 0.002
0.0361	327 ± 1 (393 ± 13)	66 ± 12	0.0129 ± 0.0003	0.423 ± 0.002
0.0480	319 ± 12 (374 ± 19)	55 ± 7	0.0131 ± 0.0009	0.411 ± 0.015
0.0550	304 ± 1 (348 ± 3)	44 ± 2	0.0135 ± 0.0005	0.392 ± 0.001
0.0654	300 ± 1 (352 ± 2)	52 ± 1	0.0138 ± 0.0012	0.387 ± 0.001
0.0725	280 ± 2 (308 ± 6)	28 ± 4	0.0142 ± 0.0002	0.362 ± 0.002
0.0849	273 ± 1 (343 ± 2)	70 ± 1	0.0152 ± 0.0010	0.352 ± 0.001
0.0956	264 ± 8 (359 ± 14)	95 ± 6	0.0153 ± 0.0007	0.340 ± 0.010
0.1184	271 ± 0 (369 ± 9)	98 ± 9	0.0182 ± 0.0027	0.349 ± 0.000

Values are mean ± standard deviations calculated over the steady state for each dilution rate. In parenthesis, total evolution (net oxygen evolution plus respiration)

Oxygen evolution and respiration rates changes in response to the different *D* were analyzed (Table 3). The net oxygen evolution rate of *Synechocystis* cells ranged between 270 μmol O_2_ mg chl^−1^ h^−1^ at the highest *D*, to more than 300 μmol O_2_ mg chl^−1^ h^−1^, in cells grown at lower *D* (between 0.0173 and 0.0654 h^−1^). Comparing the lowest and highest *D* values, respiration rates increased 6.5× times (Table 3). Respiration increased at high dilution rates, where light availability per cell was higher. Consequently, the mean gross oxygen evolution (net oxygen evolution plus respiration) did not differ much at different *D* remaining close to 352 ± 23 μmol O_2_ mg Chl^−1^ h^−1^.

### Biomass characterization

The chemical biomass composition of the cultures was determined in order to analyze its relationship with dilution rates.

#### Elemental analysis

Additional file 3: Table S2 shows the elemental composition (% DW) of the dried biomass of *Synechocystis* cells harvested at the steady-state at each *D*. The carbon and hydrogen contents were higher at low *D*, due to a greater percentage of carbohydrates in the biomass: they ranged from 42.1 ± 0.7 to 47.6 ± 0.1 % and from 6.5 ± 0.1 to 7.3 ± 0.2 % of DW, respectively. Nitrogen and sulfur contents remained fairly stable, between 10.0 ± 0.2 and 11.1 ± 0.4 % and between 0.362 ± 0.001 and 0.442 ± 0.014 %, respectively.

#### Lipid, carbohydrate and protein contents

Lipid content in the biomass (% DW) was similar at all *D* (Table 4): the average value was 11.2 ± 0.6 %. Protein content decreased slightly as *D* increased, ranging from 60.7 ± 1.1 to 72.6 ± 0.6 % with an average value of 65.0 ± 3.6 %. Amino acid profile of cells grown at three different dilution rates (0.0173, 0.0654 and 0.1184 h^−1^) did not show relevant changes (Additional file 4: Table S3). Carbohydrate content ranged from 9.4 ± 0.2 to 13.2 ± 0.2 %, with an average of 11.2 ± 1.4 %. The average ash content was 5.44 ± 0.52 %.

**Table 4:** Lipid, carbohydrate, protein, phycocyanin (Pc), allophycocyanin (Apc) and chlorophyll a (Chl) contents of *Synechocystis* dried biomass, cultured at different dilution rates

*D* (h^−1^)	Lipids (%)	Carbohydrates (%)	Proteins (%)	Pc (%)	Apc (%)	Chl (%)
0.0173	11.5 ± 0.2	11.0 ± 1.1	66.1 ± 2.5	19.6 ± 1.3	5.12 ± 029	2.33 ± 0.06
0.0198	11.6 ± 1.7	13.2 ± 0.2	62.7 ± 0.8	18.9 ± 1.1	4.56 ± 0.14	2.27 ± 0.09
0.0237	11.6 ± 1.6	12.9 ± 0.4	71.2 ± 1.5	19.2 ± 0.2	4.26 ± 0.21	2.29 ± 0.09
0.0282	12.0 ± 0.6	11.7 ± 0.7	64.7 ± 7.0	18.1 ± 0.8	3.97 ± 0.02	2.11 ± 0.04
0.0361	10.6 ± 0.9	12.5 ± 0.0	72.6 ± 0.6	17.9 ± 2.6	4.94 ± 0.09	2.24 ± 0.05
0.0480	11.0 ± 1.8	11.9 ± 0.6	64.8 ± 4.5	14.9 ± 1.3	2.49 ± 0.35	2.13 ± 0.14
0.0550	11.7 ± 0.6	12.5 ± 0.3	64.7 ± 1.2	15.4 ± 0.3	2.18 ± 0.20	2.11 ± 0.08
0.0654	11.7 ± 0.2	9.5 ± 1.0	61.2 ± 7.4	13.0 ± 1.0	1.95 ± 0.76	1.64 ± 0.14
0.0725	11.9 ± 0.2	10.7 ± 0.6	65.0 ± 2.1	13.0 ± 2.1	1.92 ± 0.99	1.84 ± 0.02
0.0849	10.5 ± 0.6	9.4 ± 0.2	64.0 ± 3.7	10.3 ± 0.9	1.63 ± 0.49	1.60 ± 0.11
0.0956	10.6 ± 0.3	10.2 ± 0.4	60.7 ± 1.1	10.9 ± 1.0	2.10 ± 0.35	1.48 ± 0.07
0.1184	10.4 ± 0.4	9.6 ± 0.7	62.2 ± 0.6	11.3 ± 1.4	2.12 ± 0.40	1.22 ± 0.18

Values are mean ± standard deviations calculated during the steady state at each dilution rate

#### Photosynthetic pigments

**Fig. 3:**
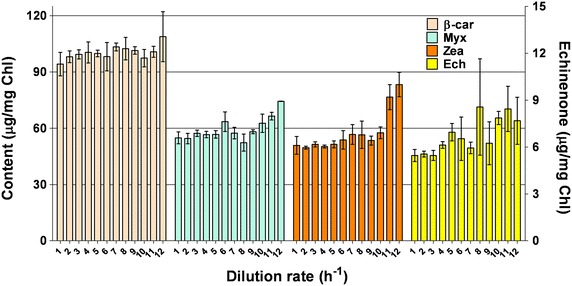
Evolution of β-car, Myx, Zea and Ech over the various dilution rates: (*1*) 0.0173 h^−1^, (*2*) 0.0198 h^−1^, (*3*) 0.0237 h^−1^, (*4*) 0.0282 h^−1^, (*5*) 0.0361 h^−1^, (*6*) 0.048 h^−1^, (*7*) 0.055 h^−1^, (*8*) 0.0654 h^−1^, (*9*) 0.0725 h^−1^, (*10*) 0.0849 h^−1^, (*11*) 0.0956 h^−1^, (*12*) 0.1184 h^−1^

Phycocyanin (Pc) and allophycocyanin (Apc) contents were highest at the lowest *D*, and decreased by 42.5 and 58.6 %, respectively, at maximum *D* (Table 4). The major carotenoids found in *Synechocystis* cells at the various *D* values were β-carotene (β-Car), myxoxanthophyll (Myx), zeaxanthin (Zea) and echinenon (Ech). Increases of 40.6 % in Ech and of 63.8 % in Zea were observed between minimum and maximum D, while the Myx increase was slightly lower (35.5 %) (Fig. 3). The chlorophyll a content decreased linearly as the *D* increased. The overall decrease was 47.6 % at the highest *D* value. On the contrary, cells grown at low *D* showed a higher chlorophyll content.

#### Biomass yield on light energy

The biomass yield (*Y*) on light energy indicates the ability of photosynthetic microorganisms to use the light energy supplied for purposes of biomass formation. In general, the energy requirement of a light-limited culture with no dark zones can be expressed by the sum of two terms [11, 12]:

Energy absorbed = Energy for biomass formation + energy required for maintenance

1 $$\phi {\text{PFD}} = \mu \times X \times V/Y_{G} + m \times X \times V$$

Hence,

2 $$(\phi {\text{PFD}} \times A)/(X \times V) = \mu /Y_{G} + m$$

where the term on the left side is the specific light absorption rate $({\text{qL}} = \phi {\text{PFD}} \times A/X \times V).$

The actual biomass yield (Y), based on the biomass production rate (g L^−1^ h^−1^), and the light-absorbed rate (*ɸPFD*) can be calculated by the following Eq. (3)

3 $$Y = X \times \mu \times V/(\phi {\text{PFD}} \times A \times 3600 \times 10^{ - 6} ) \, ({\text{g mol photons}}^{ - 1} )$$

The specific light rate absorbed by the culture layer is given by:

4 $$qL = \phi PDF \times A/(X \times V \times 3600 \times 10^{ - 6} ) \, \times \,({\text {mol photons g}}^{ - 1}\, {\rm h}^{ - 1} )$$

To assess the incidence of the maintenance energy requirement on the yield, we plotted our data following Pirt’s model [12]. This model is based on a constant light energy requirement for maintenance, as well as on a constant energy requirement for the biomass formation, i.e.:

5 $${\text{qL}} \times 0.0036 = \mu /Y_{G} + m \, ({\text{mol photons}}\, {\text{g}}^{ - 1} \,{\text{h}}^{ - 1} )$$

**Fig. 4:**
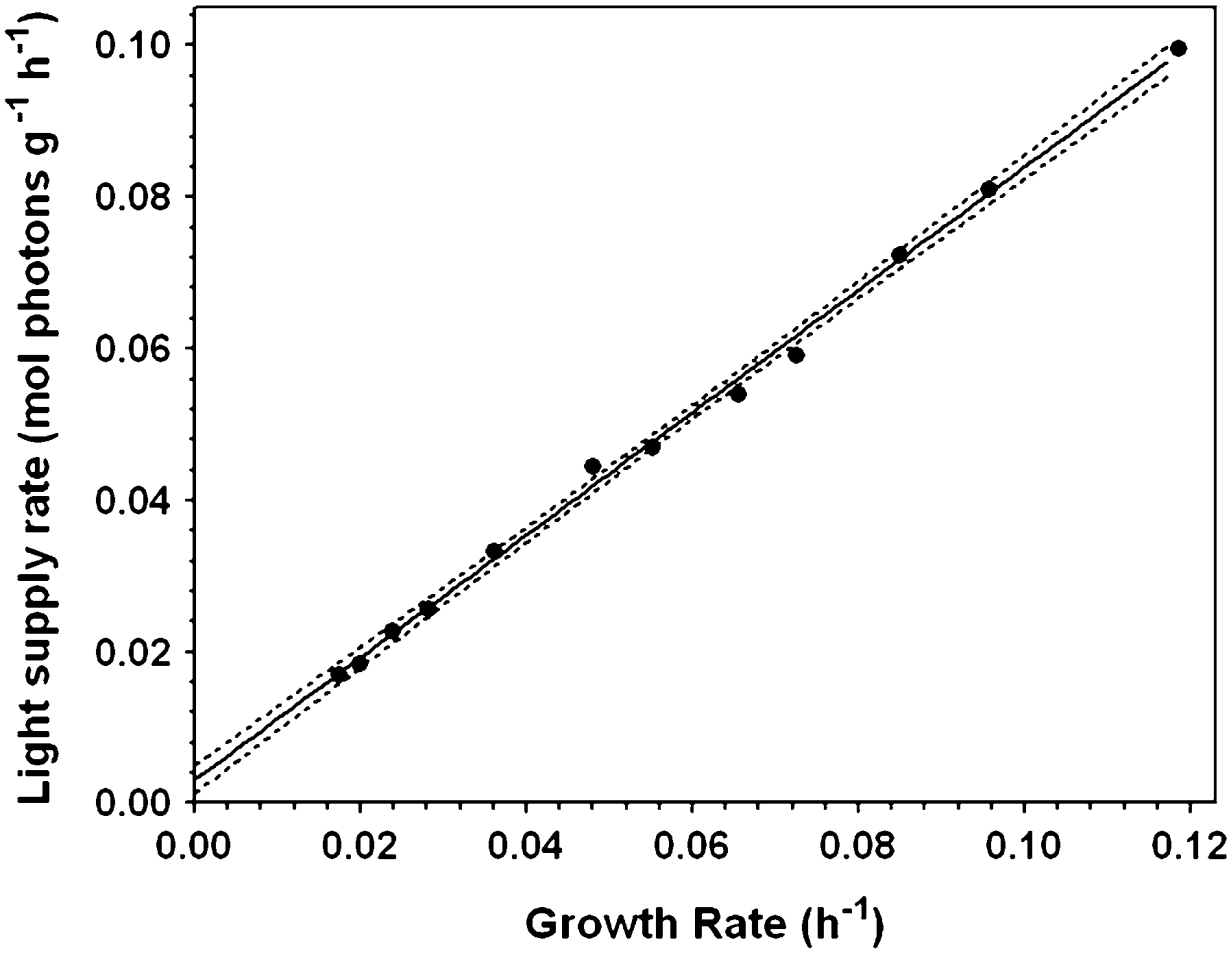
Specific light-absorbed rate by the culture as a function of growth rate. The *dashed lines* delimit the 95 % confidence intervals

By plotting the specific light-absorbed rate qL, i.e., the left-hand side of Eq. (5), versus the growth rates (*µ*) attained at the different biomass concentrations, a straight line should be obtained. The slope of this equation represents the inverse of the *Y*_G_ (maximum growth yield), and the intercept on the ordinate axes represents the maintenance coefficient (*m*) (mol photons g^−1^ h^−1^). As can be seen in Fig. 4, both the yield and the maintenance coefficient requirement were constant. The following relation was found for *Synechocystis*:

$${\text{qL}} \times 0.00 3 6 = \mu / 1. 2 3 7 + 0.00 3 1 1 7\,\,\left( {R^{ 2} = 0. 9 9 7 4} \right) \, \left( {{\text{mol photons g}}^{ - 1 } \,{\text{h}}^{ - 1} } \right)$$

Based on Pirt’s model, a constant biomass yield on the absorbed light energy of 1.237 g mol photons^−1^ was calculated for *Synechocystis*. This yield value was found to be about 35 % lower than the theoretical one (Additional file 5: Text S1).

## Discussion

Since there is considerable interest throughout the world in exploring *Synechocystis* as both an organism suitable for producing hydrogen and as a source of bioplastic material, an assessment of its photosynthetic efficiency is important. To determine the optimum growth conditions of *Synechocystis*, continuous culture experiments were carried out. Different dilution rates were applied to establish the optimal cell concentration and the maximal light conversion attainable by this organism.

A constant biomass yield on the absorbed light energy of 1.237 g mol photons^−1^ was calculated for *Synechocystis* which was considerably higher than the ones reported by Zijffers [13] for *Chlorella sorokiniana* (0.78 g mol photons^−1^) and *Dunaliella tertiolecta* (0.75 g mol photons^−1^). This discrepancy can be explained by the fact that absorbed light was used in our calculation, instead of total incident light. Another important difference was that, in our experiment, the incident light intensity was 1/6 of the amount used by the said authors, and therefore our culture might have dissipated a very small amount of energy via NPQ.

Based on Pirt’s model, LCE (PAR basis) yielded a maximum value of 12.5 %. This value was around 40 % lower than the theoretical one (i.e., 18.95 %, Additional file 5: Text S1). The discrepancy between actual and theoretical LCE was very close to that calculated for the quantum yield of photosynthesis, *Φ*_CO2_ and to the maximum quantum yield of PSII (*F*_v_/*F*_m_). This ratio was in fact lower than the one normally reported in higher plants and microalgae (0.75–0.85). It is conceivable that the photosynthetic apparatus of *Synechocystis* cells it normally stays in state 2, which is characterized by lower fluorescence and photosynthesis rates and high energy dissipation via state transition quenching (qT) [14]. qT is attributed to the decoupling of phycobilisomes from PSII [14]. It has been reported that phycobilisome decoupling is seemingly important not only under strong irradiation [15], but also at physiological conditions of irradiance, provided that the exposure time is sufficiently long. It has been suggested that the dissipation of light energy in *Synechocystis* may occur via both the orange carotenoid protein (OCP) [16] in blue actinic light and a rise in orange light-induced S, M fluorescence [14].

The actual biomass yield (*Y*, g mol photons^−1^) was relatively constant at most of the high *D* (Table 1). Between minimum and maximum *D* values, an increase in *Y* of about 20 % was observed. This could be attributed to the greater availability of light and to the higher growth yield *Y*. The highest biomass yield on light energy (1.22 ± 0.05 g mol photons^−1^) was attained at *D* = 0.0725 h^−1^.

LCE values at different *D* were fairly constant (mean 11.3 ± 0.41 %). Most likely, the efficient mixing system, consisting of a specially designed rotating impeller [17], and the absence of any dark zones in the reactor, prevented cells from becoming acclimated to low light even when they were grown at low *D* (dense cultures). On the other hand, cells grown at a high *D* reacted by reducing the chlorophyll antenna and increasing their optical absorption cross section, thus making better use of light per chlorophyll unit. In our culture system the photon flux uptake ranged from 16 to 95 mmol photons g^−1^ h^−1^, with optimum value close to 50 mmol photons g^−1^ h^−1^, that is, in correspondence to the highest LCE attained. These findings agree with the metabolic model proposed by Nogales et al. [18].

Both the maximum photochemical quantum yield (*F*_v_/*F*_m_) and the effective quantum yield of PSII (*∆F*/F_m_^′^) declined by about 20 % between minimum and maximum *D*. This occurrence was also accompanied by a slight increase in *V*_J_ of the chlorophyll fluorescence transient. A lower decrease in the ETR_max_ was recorded (about 10 % at the extreme *D*). This lower difference compared to that observed in the effective quantum yield of PSII (∆*F*/*F*_m_^′^) could be explained by the fact that with the increase of *D* the cells reacted with a 40 % increase in the chlorophyll optical absorption cross section. Despite the reduction in the *F*_v_/*F*_m_ ratio, gross photosynthesis (i.e. net photosynthesis plus respiration) did not show important changes and was found to be almost independent of *D*. Reductions in the net photosynthesis rate was compensated by a proportional increase in the respiration rate, which increased with increasing *D*. The apparent paradox of a reduction in the *F*_v_/*F*_m_ ratio without a parallel reduction in the maximum photosynthesis rate had already been reported by Behrenfeld et al. [19].

Quantum yield *Φ*_CO2_ determined in this study resulted higher (close to 0.06 mol CO_2_ mol photons^−1^) in cells grown at lower *D* (between 0.0173 and 0.0282 h^−1^). This value was close to that reported by Skillman [20] for C4 plants and that calculated by Nogales et al. using cyanobacteria system analysis [21]. The value slightly declined with the increase of *D*, which reduced the cell concentration and therefore led to a higher exposure of cells to light and consequently to a reduction in the effective quantum yield of PSII. Yet, these changes were not detected by changes in NPQ which was most likely underestimated due to the difficulty of correctly measuring *F*_m_ in dark adapted cultures of cyanobacteria. Moreover, in our calculation we assumed that PSII/PSI ratio was constant and equal to 1. This ratio is close to the value reported by Fujimori et al. [22], who used *Synechocystis* cultures exposed to a constant light intensity similar to our experiments. However, PSII/PSI may range from 0.4 to 1.0 from low light-adapted to high light-adapted cultures [22, 23]. Much higher changes in the PSII/PSI ratio can be expected in carbon limited *Synechocystis* cultures as reported by Nogales [21]. PSII/PSI as low as 0.2 has been reported [24]. This unusual stoichiometry in cyanobacteria has been explained with the involvement of PSI in a significant amount of cyclic electron flow around this photosystem. Alternatively, high PSI amount in cyanobacteria may serve to balance the abundance of the respiratory electron transfer pathways into the PQ pool [25]. Both hypothesis are suggested to act as photoenergy-dissipation pathways [21, 25]. About 20 % of electrons originated from water was targeted to O_2_ via Mehler reaction (water–water cycle) in wild type *Synechocystis* grown under atmospheric CO_2_ levels [26, 27].

## Conclusions

The limited amount of available fossil energy on the one hand and the necessity: of gearing the economy towards low carbon emissions on the other, intensify the demand for clean energy sources for the near future. *Synechocystis* is a model system: thanks to its available genomic sequence and to its ability to be naturally transformable. It could provide renewable energy in the form of hydrogen, which is considered to be the most important energy carrier [3, 28]. Moreover, its use has been proposed for photosynthetic production of isoprene for various applications, such as the production of rubber, adhesives, plastics, and perfumes [5–7].

This study provides a significant data set analyzing the maximum threshold of LCE as well as basic bioenergetic parameters of high-density cultures of *Synechocystis*, a microorganism of immense biotechnological potential. Our experiments will help future attempts of genetic transformation of this organism and improve in silico analysis of cyanobacterial photosynthetic models.

We would like to emphasize that the mean effective LCE attained by *Synechocystis* in this study (11.32 ± 0.41 %) was under strictly controlled laboratory conditions (refers to PAR from 400 to 700 nm). Under more natural outdoor conditions with variable environmental factors (e.g., temperature, mixing, nutrients), the LCE, typically drops to 5–6 % of total solar radiation, of which PAR is assumed to account for 45 %. Similar LCE was also found by Zhu et al. for higher plants [29]. The culture studied in our lab was exposed to a light intensity which was roughly 1/10th of that recorded outdoors on a typical day in summer. Therefore, it would be unrealistic to compare laboratory performance of *Synechocystis* to outdoor culture scenarios.

## Methods

### Photobioreactor set-up

**Fig. 5:**
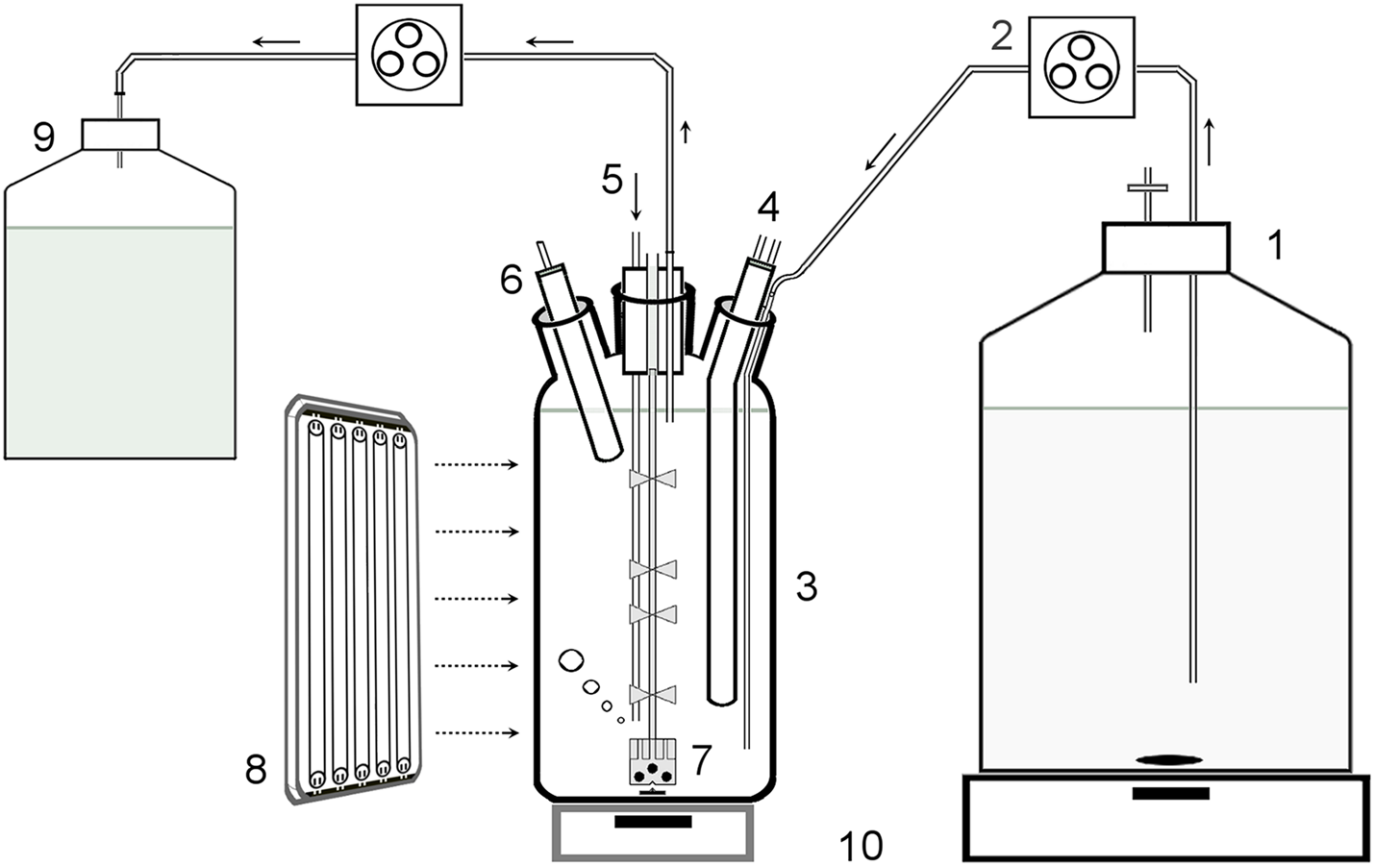
Schematic representation of the experimental set-up for the continuous culture of *Synechocystis*. (*1*) medium inlet bottle, (*2*) peristaltic pump, (*3*) Roux-type bottle, (*4*) cooling finger, (*5*) Air/CO_2_ inflow, (*6*) pH sensor, (*7*) impeller, (*8*) lamp panel, (*9*), harvest bottle, (*10*) magnetic stirrers

The system consisted of a 1 L Pyrex Roux-type culture bottle (950 mL working volume, 5 cm light path) with a flat cross section (12 × 5 cm width), a flat bottom, and four ports for the pH meter electrode, the medium inlet, the air/CO_2_ inlet, and the cooling finger (Fig. 5). The main port at the top (2.5 cm i.d.) was sealed with a stopper equipped with tygon tubes for the outflow of culture and gases. Illumination was provided by means of cool white lamps (Dulux L, 55 W/840, Osram, Italy) with a photon flux density (PFD) of 150 μmol photons m^−2^ s^−1^. The PFD at the culture surface was measured with a flat quantum radio-photometer (LI-250A, LI-COR Biosciences). Mixing of the culture was achieved by means of a specially designed rotating impeller [17] driven magnetically by a stirrer at the bottom. A constant air flow was provided for the removal of oxygen, and a pH value of 7.4 was maintained by automatic addition of CO_2_. The air and CO_2_ were sterilized using 0.2 μm pore size filters (Whatman, UK). The culture’s temperature was maintained constant at 28.0 ± 0.2 °C with the use of an external thermostat (MPM Instruments—Italy). For the medium inlet/outlet, two peristaltic pumps were used (Masterflex 7520-30 Cole Parmer for the medium inlet and SP311 Velp Scientifica for the medium outlet). The culture was assumed to be at a steady state when the cell concentration assessed with the dry weight method remained unchanged for at least 36 h.

### Strains and medium

The wild type *Synechocystis* strain PCC6803 was kindly provided by Prof. M. Rӧgner (Plant Biochemistry, Ruhr University Bochum, Germany). It was pre-cultured in a BG-11 medium under artificial light (intensity: 35 μmol photons m^−2^ s^−1^) supplied from one side of the glass tube (cultivation columns) (i.d. = 50 mm; 400 mL working volume). The cultures were bubbled with a mixture of air-CO_2_ (97/3 v/v) at a continuous flow rate of 5 dm^3^ min^−1^. The growth was measured daily with the dry weight method.

### Culture characterization

In a continuous culture system, cell growth is controlled by a continuous inflow of fresh sterile medium. The flow of fresh medium through the system is defined as dilution rate *D* = *F*/*V*, where *F* (mL L^−1^ h^−1^) is the flow rate of the fresh medium and *V* (L) is the culture volume. The change in biomass (*x*, g L^−1^) over time can be described as d*x*/d*t* = growth-output, and d*x*/d*t* = μ*x* − *Dx*, where *μ* (h^−1^) is the specific growth rate.

When the cell concentration remains constant over time (steady state), i.e., d*x*/d*t* ≈ 0, then *μ* = *D*. The doubling time *t*_*d*_ (h) of the cells is given by the following equation: *t*_*d*_ = ln2/μ.

Productivity *P* (mg h^−1^) in continuous cultivation is dependent on the concentration of biomass (*X*), and also on the dilution rate (*D*). At the steady state, productivity is calculated as *P* = *μXV*.

Dry weight (DW) was determined in duplicate by using 10 mL samples taken from the PBR every day. Each sample was filtered through a pre-weighted 47 mm diameter glass microfiber filter membrane (Whatman GF/F filters, Maidstone, England). The cells were washed twice with deionized water the filters were then oven-dried at 105 °C to a complete evaporation (usually within 3 h) and weighed. Lyophilized biomass was obtained from frozen samples (−40 °C) using a 5PASCAL LIO10P instrument (24 h at 0.1 mbar). The elemental composition analysis of the biomass was performed using a CHNOS Analyzer, Flash EA, 1112 Series (Thermo Electron Corporation). The ash content was determined by heating the biomass at 450 °C for 24 h.

### Fluorescence measurements

Chlorophyll a fluorescence transients were recorded using a Handy PEA (Hansatech Instruments, UK) in 2 mL dark adapted samples that were illuminated with continuous light (650 nm peak wavelength, 3500 μmol photons m^−2^ s^−1^) provided by light-emitting diodes (LEDs). Each chlorophyll a fluorescence induction curve was analyzed using “BiolyzerHP3” software. The following parameters were calculated from the fluorescence measurements:

- minimum fluorescence yield, *F*_0_, recorded at 50 μs;
- maximum fluorescence yield, *F*_m_;
- *M*_0_ = 4(*F*_300μs_ − *F*_0_)/(*F*_m_ − *F*_0_), which corresponded to the net rate of the reaction center closure, where it increases by means of trapping and decreases by means of electron transport;
- the variable fluorescence at phase J, *V*_J_ = (*F*_J_ − *F*_0_)/(*F*_m_ − *F*_0_), which is considered to be a good indicator of the plastoquinone pool redox state;
- the maximum quantum yield of photosystem II (PSII) for primary photochemistry, *Φ*(*P*_0_), calculated as *F*_v_/*F*_m_ = (*F*_m_ − *F*_0_)/*F*_m_;
- the quantum yield of the electron transport *Φ*_E0_, *Φ*_E0_ = *Φ*(*P*_0_) × *Ψ*_0_, where *Ψ*_0_ is the efficiency with which a trapped exciton can move an electron further than *Q*_A_^−^ (primary quinone electron acceptor) into the electron transport chain;
- the parameter *S*_m_, a measurement of the energy needed to reduce *Q*_A_ completely, was calculated by dividing the area by *F*_v_.

Slow kinetic analysis of chlorophyll fluorescence was performed using a pulse-amplitude-modulation fluorometer (PAM-2100, H. Walz, Effeltrich, Germany) operated by means of PamWin (version 2.00f) PC software. The ratio between variable and maximum fluorescence, *F*_v_/*F*_m_, was then measured to determine the maximum photochemical yield of PSII. For this purpose, samples were taken from the PBR and incubated in the dark for 15 min to remove any energy-dependent quenching. In addition, one far-red light (above 700 nm) pulse with a duration of 10 s (10 W m^−2^), supplied by the PAM-2100, was applied. For comparison, measurements of *F*_v_/*F*_m_ were also performed in the light using 3-(3,4-dichlorophenyl)-1,1-dimethylurea (DCMU) (10^−5^ M), and resulted in a lower value, therefore all the measurements were carried out under far-red light. The photochemical quantum yield of PSII in the light-adapted state, i.e., when the PSII reaction centers are open (*F*_v_^′^/*F*_m_^′^), was calculated by using the variable fluorescence (*F*_v_^′^ = *F*_m_^′^ − *F*_0_^′^) and the maximum fluorescence from the light-adapted culture (*F*_m_^′^). *F*_*v*_^′^/*F*_m_^′^ indicates the proportion of light-absorbed by PSII that is potentially usable for photochemistry in the light. The effective photochemical quantum yield of PSII Δ*F*/*F*_m_^′^ = (*F*_m_^′^ – *F*_s_)/*F*_m_^′^, which is the number of electrons generated per photon absorbed [30], was measured using *F*_s_ and *F*_m_, which represented the steady-state and maximum fluorescence measured in the light. *F*_s_ and *F*_m_^′^ were determined by putting the fiber-optic probe of the fluorometer directly in contact on the illuminated PBR surface at an angle of 60°. Non-photochemical quenching (NPQ) was calculated by using the Stern–Volmer equation NPQ = (*F*_m_ – *F*_m_^′^)/*F*_m_^′^ [31]. *F*_0_^′^ was estimated from the following relationship: *F*_0_^′^ = *F*_0_/[*F*_v_/*F*_m_ + *F*_o_/*F*_m_^′^] [32]. The photochemical quenching (qP) was calculated according to Kromkamp and Forster [33].

### Cross section and electron transport rate

The average chlorophyll specific optical absorption cross section (a*) of the cells was determined according to Kromkamp and Limbeek [34] from in vivo absorption spectra (range 400–750 nm) recorded on a Varian Cary50 UV–visible spectrophotometer. To minimize the impact of the light scattering effect from the cells’ surface on the absorbance measurement, the sample in the cuvette was positioned right next to the detector window.

The photosynthetic electron transport rate of PSII (ETR, μmol e^−^ mg chl^−1^ s^−1^) was estimated from the following relationship:

$${\text{ETR}} = \Delta F/F_{\text{m}}^{\prime} \times {\text{PAR}} \times a^{*} \times 0. 5$$

assuming that the photosystem I (PSI) to PSII (PSI/PSII) ratio of *Synechocystis* PCC6803 is 1 according to Fujimori et al. [22]. Thus, 50 % of the photons are absorbed by PSII [35], and a* (m^2^ mg chl^−1^) was the average chlorophyll optical cross section normalized to chlorophyll a. This equation assumes that no cyclic electron transport by PSI occurs.

The quantum yield of CO_2_ assimilation, *Φ*_CO2_, was calculated from the product of *Φ*PSII × fraction PSII × (1/4) [36] where *Φ*_PSII_ = Δ*F*/*F*_m_^′^.

### Oxygen evolution measurements

Oxygen evolution measurements were carried out in triplicate on 2 mL culture samples (chlorophyll content: 5 mg L^−1^), using a Liquid-Phase Oxygen Electrode Chamber (Hansatech, DW3) thermostated at 28 °C and equipped with an oxygen control electrode unit (Hansatech, Oxy-lab). Light was supplied via a red LED light source (Hansatech LH36/2R) at 637 nm wavelength providing a 500 μmol photons m^−2^ s^−1^ PFD. The O_2_ concentration dissolved in the sample was continuously monitored at an acquisition rate of 0.2 reads s^−1^. Dark respiration rates were measured after the photosynthesis rates had been measured.

### Chlorophyll and carotenoid analysis

Chlorophyll concentration was determined spectrophotometrically in triplicate samples. 5 mL samples were centrifuged in glass tubes for 8 min at 2650*g* in an ALC-PK110 centrifuge. The supernatant was discarded, and the pellet was resuspended in 5 mL of pure methanol. The tubes were then placed in a 70 °C water bath for 3 min, and centrifuged again for 8 min at 2650*g*. The supernatant absorbance was measured at 665 and 750 nm against a pure methanol blind. The concentrations of individual carotenoids were assessed using a reversed-phase Beckman System Gold HPLC (module 125 solvent) equipped with a diode array detector, model 168 Nouveau (Beckman Instruments, Inc., CA, USA), with a column Luna, C8 (Phenomenex), in accordance with Van Heukelem and Thomas [37].

### Protein, carbohydrate and lipid contents

Protein determination was performed according to Lowry [38]. The total carbohydrate content was measured using the phenol–sulfuric acid method [39], with *D*+ glucose used as the standard. Lipids were extracted from 5 mg of dry biomass using 1 mL of dichloromethane, 2 mL of methanol and 0.8 mL of deionized water (1:2:0.8, v/v/v). The mixture was vortexed and sonicated for 10 min, after which an additional 1 mL of dichloromethane and 1 mL of deionized water were added. The mixture was then vortexed and centrifugated for 5 min at 1500*g* (ALC-PK110). The bottom phase was recovered, placed in pre-weighted containers, and heated to complete evaporation. The extracted lipids were then weighed. The analyses were performed in triplicate. The heat of combustion (kJ g^−1^) of the biomass at the steady-state of each *D* was calculated by using the formula:

$$\begin{aligned} & ( [ ( {\text{proteins}} \times 5. 7 ) + ( {\text{carbohydrates}} \times 4. 2 ) \\ & \quad + ( {\text{lipids}} \times 9. 3 ) ]/ 100 ) \times 4. 1 8 4.\end{aligned}$$

Amino acid composition was determined according to Potenza et al. [40].

### Phycocyanin extraction and determination

Culture samples (5 mL) were collected in tubes and centrifuged at 2650*g* for 8 min. The supernatant was discarded, and 0.5 mL of glass beads (diameter 0.17–0.18 mm, B. Braun Biotech Int—Germany) were added to the sample, along with 200 μL of NaCl 0.15 M phosphate-buffered (pH 7.4) solution. The mixture was vortexed for 10 min to break down the cells; thereafter, phosphate buffer was then added to reach 5 mL of final volume. The tubes were centrifuged at 2650*g* for 5 min, the supernatant was then transferred into 15 mL Falcon tubes and centrifuged at 12500*g* for 10 min. The absorbance values at 652 nm (allophycocyanin) and 615 nm (phycocyanin) were measured, after which the concentrations of allophycocyanin and phycocyanin were calculated according to Bennett and Bogorad [41]. The measurements were performed in duplicate.

## Additional files

Additional file 1:
**Figure S1.** The maximum quantum yield of PSII (F_v_/F_m_) and ΔF/F_m_’ of *Synechocystis* as a function of the dilution rate. Additional file 2:
**Table S1.** The M_0_ (initial slope at the beginning of the variable fluorescence), V_J_ (the variable fluorescence at phase J), Φ_E0_ (the quantum yield of electron transport), Ψ_0_ (the efficiency with which a trapped exciton can move an electron further than Q_A_^-^ into the electron transport chain) and the parameter S_m_ of *Synechocystis* in a continuous culture. Values are mean ± standard deviations calculated over the steady state for each dilution rate. Additional file 3:
**Table S2.** Elemental composition of *Synechocystis* dried biomass, cultured at different dilution rates. Values are mean ± standard deviations calculated during the steady state at each D (h^-1^). Additional file 4:
**Table S3.** Comparison of amino acid composition of *Synechocystis* cultured at three different dilution rates. Additional file 5:
**Text S1.** Theoretical biomass yield on light energy calculation.

## Abbreviations

A: illuminated reactor surface area (m^2^)
X: biomass concentration (g L^−1^)
D: dilution rate (h^−1^)
E: mean energy content of one mol of photons in the visible spectra (kJ mol photons^−1^)
LCE: light conversion efficiency (-)
M: biomass maintenance coefficient (mol photons g^−1^ h^−1^)
PAR: photosynthetic active radiation (µmol photons m^−2^ s^−1^)
qL: specific rate of absorption of radiation (mol photons g^−1^ h^−1^)
µ: growth rate (h^-1^)
V: volume of the bioreactor (L)
Y: actual biomass growth yield on light-absorbed energy (g mol photons^−1^)
Y_G_: maximum attainable biomass growth yield on light-absorbed energy (g mol photons^−1^)
ɸ: fraction of PAR absorbed by cultures (−)
AEF: alternative electron flow
PQ: plastoquinone pool
