# Supplementary figures and images for: A bioenergetic assessment of photosynthetic growth of Synechocystis sp. PCC 6803 in continuous cultures
Source: Biotechnol Biofuels. 2015 Sep 4;8:133. doi: 10.1186/s13068-015-0319-7 (PMC4571542; doi:10.1186/s13068-015-0319-7)

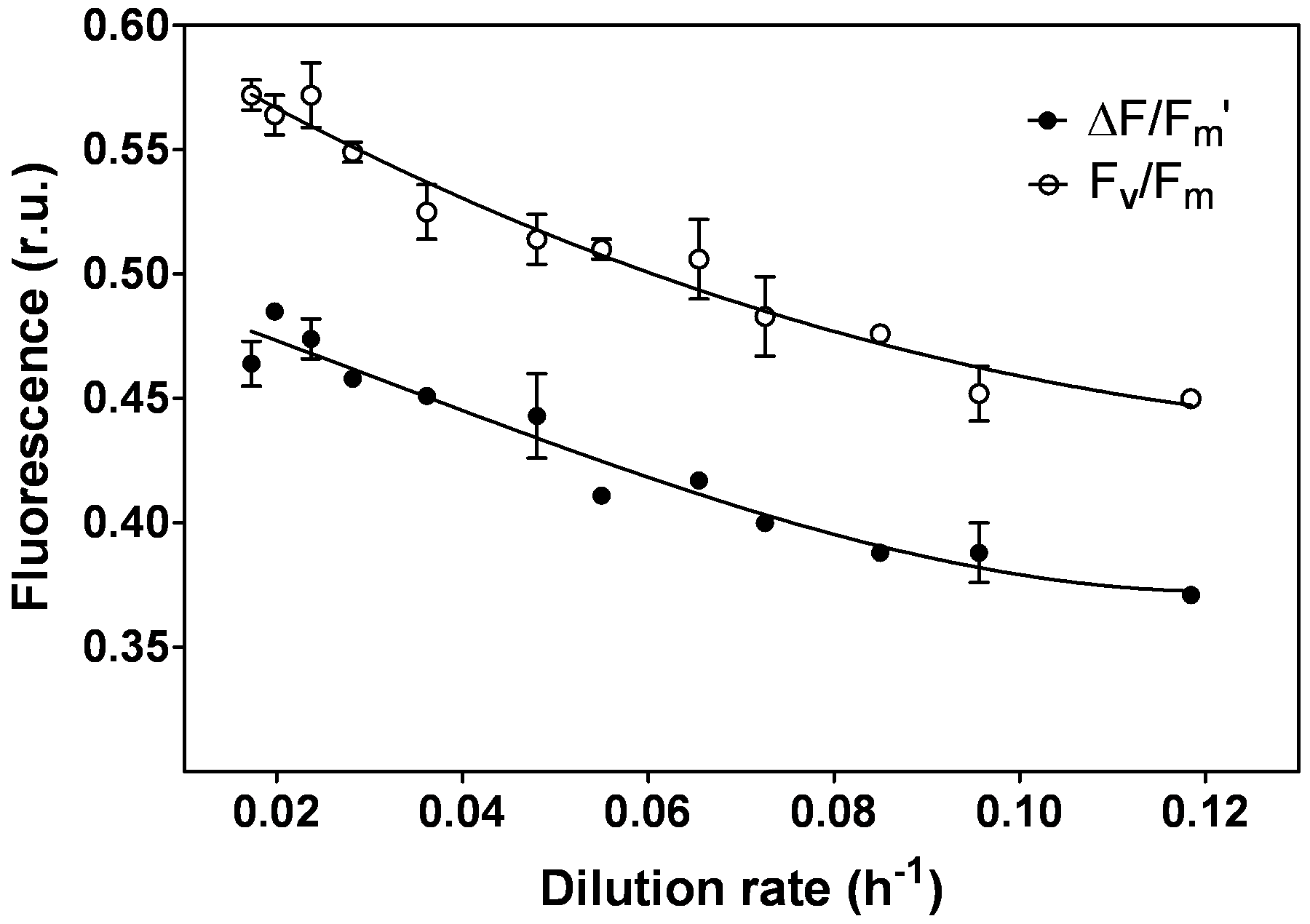

Supplement: Additional file 1: — Figure S1. The maximum quantum yield of PSII (Fv/Fm) and ΔF/Fm’ of Synechocystis as a function of the dilution rate. [file 13068_2015_319_MOESM1_ESM.tiff]
