# Supplementary material for: A bioenergetic assessment of photosynthetic growth of Synechocystis sp. PCC 6803 in continuous cultures
Source: Biotechnol Biofuels. 2015 Sep 4;8:133. doi: 10.1186/s13068-015-0319-7 (PMC4571542; doi:10.1186/s13068-015-0319-7)
Supplement: Additional file 2: — Table S1. The M0 (initial slope at the beginning of the variable fluorescence), VJ (the variable fluorescence at phase J), ΦE0 (the quantum yield of electron transport), Ψ0 (the efficiency with which a trapped exciton can move an electron further than QA- into the electron transport chain) and the parameter Sm of Synechocystis in a continuous culture. Values are mean ± standard deviations calculated over the steady state for each dilution rate. [file 13068_2015_319_MOESM2_ESM.doc]

| **D (h^-1^)** | **M_0_** | **V_J_** | **Ψ_0_** | **Φ_Ε0_** | **S_m_** |
| --- | --- | --- | --- | --- | --- |
| 0.0173 | 1.50±0.02 | 0.499±0.013 | 0.501±0.013 | 0.197±0.005 | 64.1±2.2 |
| 0.0198 | 1.41±0.14 | 0.472±0.059 | 0.528±0.059 | 0.216±0.021 | 64.4±3.9 |
| 0.0237 | 1.50±0.02 | 0.524±0.020 | 0.475±0.020 | 0.188±0.007 | 61.6±5.5 |
| 0.0282 | 1.49±0.03 | 0.491±0.033 | 0.509±0.033 | 0.191±0.015 | 68.2±5.6 |
| 0.0361 | 1.47±0.01 | 0.463±0.006 | 0.537±0.006 | 0.200±0.000 | 58.6±0.3 |
| 0.0480 | 1.75±0.07 | 0.542±0.035 | 0.456±0.046 | 0.150±0.011 | 56.2±4.2 |
| 0.0550 | 1.75±0.12 | 0.574±0.073 | 0.426±0.073 | 0.170±0.009 | 56.5±7.0 |
| 0.0654 | 1.84±0.04 | 0.574±0.030 | 0.425±0.030 | 0.130±0.010 | 53.3±2.7 |
| 0.0725 | 1.83±0.07 | 0.574±0.055 | 0.425±0.055 | 0.130±0.014 | 57.7±1.3 |
| 0.0849 | 1.99±0.01 | 0.572±0.034 | 0.428±0.034 | 0.124±0.004 | 57.9±6.6 |
| 0.0956 | 2.05±0.02 | 0.595±0.019 | 0.405±0.019 | 0.119±0.007 | 59.8±1.6 |
| 0.1184 | 2.14±0.07 | 0.623±0.044 | 0.377±0.044 | 0.113±0.006 | 57.7±1.6 |
