# Supplementary material for: A bioenergetic assessment of photosynthetic growth of Synechocystis sp. PCC 6803 in continuous cultures
Source: Biotechnol Biofuels. 2015 Sep 4;8:133. doi: 10.1186/s13068-015-0319-7 (PMC4571542; doi:10.1186/s13068-015-0319-7)
Supplement: Additional file 3: — Table S2. Elemental composition of Synechocystis dried biomass, cultured at different dilution rates. Values are mean ± standard deviations calculated during the steady state at each D (h-1). [file 13068_2015_319_MOESM3_ESM.doc]

| **D (h^-1^)** | **C (%)** | **N (%)** | **H (%)** | **S (%)** | **O (%)** |
| --- | --- | --- | --- | --- | --- |
| 0.0173 | 47.3±0.1 | 10.9±0.3 | 7.0±0.2 | 0.42±0.02 | 21.0±0.4 |
| 0.0198 | 47.6±0.1 | 11.1±0.1 | 7.3±0.1 | 0.44±0.01 | 21.5±0.1 |
| 0.0237 | 46.4±0.4 | 10.0±0.2 | 6.7±0.1 | 0.43±0.02 | 21.1±0.3 |
| 0.0282 | 46.7±0.5 | 11.1±0.4 | 7.3±0.2 | 0.42±0.02 | 22.5±0.3 |
| 0.0361 | 46.4±0.1 | 11.1±0.4 | 7.1±0.1 | 0.42±0.01 | 21.8±0.3 |
| 0.0480 | 46.2±0.3 | 10.7±0.2 | 7.3±0.1 | 0.44±0.04 | 22.1±0.7 |
| 0.0550 | 45.3±1.0 | 10.8±0.0 | 6.9±0.5 | 0.44±0.01 | 22.2±1.1 |
| 0.0654 | 45.3±0.6 | 10.9±0.2 | 6.6±0.1 | 0.44±0.01 | 22.3±0.4 |
| 0.0725 | 45.1±0.5 | 10.9±0.2 | 6.7±0.1 | 0.43±0.02 | 22.3±0.2 |
| 0.0849 | 44.1±0.7 | 11.1±0.1 | 6.8±0.1 | 0.42±0.00 | 23.8±0.4 |
| 0.0956 | 43.7±0.9 | 10.8±0.2 | 6.7±0.2 | 0.39±0.01 | 23.2±0.2 |
| 0.1184 | 42.1±0.7 | 10.3±0.2 | 6.5±0.1 | 0.36±0.00 | 23.6±0.4 |
