# Supplementary material for: A bioenergetic assessment of photosynthetic growth of Synechocystis sp. PCC 6803 in continuous cultures
Source: Biotechnol Biofuels. 2015 Sep 4;8:133. doi: 10.1186/s13068-015-0319-7 (PMC4571542; doi:10.1186/s13068-015-0319-7)
Supplement: Additional file 4: — Table S3. Comparison of amino acid composition of Synechocystis cultured at three different dilution rates. [file 13068_2015_319_MOESM4_ESM.doc]

|  | **Dilution rate (h^-1^)** | | |
| --- | --- | --- | --- |
| **Amino acid**  % (w/w) | **0.0173** | **0.0654** | **0.1184** |
| **His** | 1.53 | 1.45 | 1.49 |
| **Ser** | 3.58 | 3.48 | 3.50 |
| **Arg** | 9.83 | 10.00 | 10.36 |
| **Gly** | 4.91 | 5.02 | 5.16 |
| **Asp** | 12.33 | 11.93 | 12.00 |
| **Glu** | 12.69 | 12.06 | 12.77 |
| **Thr** | 4.70 | 4.56 | 4.68 |
| **Ala** | 9.18 | 9.06 | 8.91 |
| **Pro** | 4.33 | 4.46 | 4.40 |
| **Cys** | 0.05 | 0.06 | 0.07 |
| **Lys** | 4.80 | 5.26 | 5.45 |
| **Tyr** | 2.84 | 3.00 | 2.50 |
| **Met** | 1.60 | 1.66 | 1.41 |
| **Val** | 6.33 | 6.45 | 6.51 |
| **Ile** | 6.12 | 6.27 | 6.18 |
| **Leu** | 10.19 | 10.23 | 9.88 |
| **Phe** | 5.07 | 4.97 | 4.78 |
