# Supplementary material for: A bioenergetic assessment of photosynthetic growth of Synechocystis sp. PCC 6803 in continuous cultures
Source: Biotechnol Biofuels. 2015 Sep 4;8:133. doi: 10.1186/s13068-015-0319-7 (PMC4571542; doi:10.1186/s13068-015-0319-7)
Supplement: Additional file 5: — Text S1. Theoretical biomass yield on light energy calculation. [file 13068_2015_319_MOESM5_ESM.doc]

**Theoretical biomass yield on light energy calculation**

The theoretical biomass yield on light energy can be calculated based on the stoichiometric reaction equation for the formation of biomass on carbon dioxide, water, and the nitrogen source used for the growth of the organism.

For growth on nitrate:

CO_2_ (g) + H_2_O (l) + 0.203NO_3_^-^ (aq) → CH_1.82_O_0.36_N_0.203_ (s) + 1.32 O_2_ + 0.203 OH^-^ (aq), eq. (1)

The photon yield of primary photochemistry is 1 electron per absorbed photon in PSI and < 0.8 electrons per absorbed photon in PSII [1-4], therefore, the reduction of 2 NADP^+^ and production of 1 O_2_ need at least 4 + (4/8) = 9 photons. According to the elemental composition of *Synechocystis* biomass, the molecular mass of a C-mol biomass is 22.42 g mol^-1^. To synthesize one C-mol of biomass, 11.9 mol of photons are required in order to evolve the amount of O_2_, in accordance with stoichiometry reaction equations (eq. (1)). This would lead to a theoretical biomass yield of 1.88 g mol photons^-1^. Theoretical light conversion efficiency was calculated as follows: LCE = (Y_G_ kJ g^-1^)/E

The energy content of biomass (heat of combustion) was found to be 21.98 ± 1.05 kJ g^-1^, while the mean energy content of one mol of photons in the visible spectra was assumed to be 218 kJ mol photons^-1^.

Hence: LCE = (1.88×21.98)/218×100 = 18.95%.

1. Raven JA, Beardall J, Giordano M: **Energy costs of carbon dioxide concentrating mechanisms in aquatic organisms.** *Photosynth Res* 2014, **121**(2-3):111-124.
2. Hogewoning SW, Wientjes E, Douwstra P, Trouwborst G, von Ieperen W, Grace R, Harbinson J: **Photosynthetic quantum yield dynamics: from photosynthesis to leaves.** *Plant Cell* 2012, **24**:1921-1936.
3. Skllman JB: **Quantum yield variation across the three pathways of photosynthesis:not yet out of the dark.** *J Exp Bot* 2008, **59**:1647-1661.
4. Kramer DM, Evans JR: **The importance of energy balance in improving photosynthetic productivity.** *Plant Physiol* 2011, **55**:70-78.
